# RMI1 facilitates repair of ionizing radiation–induced DNA damage and maintenance of genomic stability

**DOI:** 10.1038/s41420-023-01726-1

**Published:** 2023-11-25

**Authors:** Lianying Fang, Yuxiao Sun, Mingxin Dong, Mengmeng Yang, Jianxiu Hao, Jiale Li, Huanteng Zhang, Ningning He, Liqing Du, Chang Xu

**Affiliations:** 1 Tianjin Key Laboratory of Radiation Medicine and Molecular Nuclear Medicine, Institute of Radiation Medicine, Chinese Academy of Medical Sciences and Peking Union Medical College, Tianjin, 300192 China; 2 School of Preventive Medicine Sciences, Institute of Radiation Medicine, Shandong First Medical University, Shandong Academy of Medical Sciences, Jinan, 250062 China

**Keywords:** Double-strand DNA breaks, Homologous recombination

## Abstract

Ionizing radiation (IR) causes a wide variety of DNA lesions, of which DNA double-stranded breaks (DSBs) are the most deleterious. Homologous recombination (HR) is a crucial route responsible for repairing DSBs. RecQ-mediated genome instability protein 1 (RMI1) is a member of an evolutionarily conserved Bloom syndrome complex, which prevents and resolves aberrant recombination products during HR, thereby promoting genome stability. However, little is known about the role of RMI1 in regulating the cellular response to IR. This study aimed to understand the cellular functions and molecular mechanisms by which RMI1 maintains genomic stability after IR exposure. Here, we showed IR upregulated the RMI1 protein level and induced RMI1 relocation to the DNA damage sites. We also demonstrated that the loss of RMI1 in cells resulted in enhanced levels of DNA damage, sustained cell cycle arrest, and impaired HR repair after IR, leading to reduced cell viability and elevated genome instability. Taken together, our results highlighted the direct roles of RMI1 in response to DNA damage induced by IR and implied that RMI1 might be a new genome safeguard molecule to radiation-induced damage.

## Introduction

Cells are constantly under genotoxic pressure from the environment and internal metabolic processes, posing a serious threat to the maintenance of genome stability [1]. Genomic instability is one of the main causes of various diseases such as cancer, immune deficiency diseases, and neurodegenerative diseases [2]. Ionizing radiation (IR) is a high-energy radiation that produces intermediate ions and free radicals to damage genetic materials, leading to genomic instability [3–5]. DNA double-stranded breaks (DSBs) are one of the most threatening DNA lesions among various types of IR-induced DNA damage [6, 7]. In eukaryotic cells, DSBs are primarily repaired through non-homologous end joining (NHEJ) and homologous recombination (HR) [8]. The functions of HR and NHEJ are strictly regulated throughout the cell cycle, and the appropriate choice between these two repair pathways is essential for DSB repair and maintenance of genomic stability [9]. HR repair employs mostly homologous DNA in the sister chromatid as a template, restoring the correct DNA sequence. Consequently, this repair mechanism predominantly occurs during the S and G2 phases of the cell cycle [10].

Bloom DNA helicase (BLM) combines closely with DNA topoisomerase IIIα (Top3A), RecQ-mediated genome instability protein 1 (RMI1), and RecQ-mediated genome instability protein 2 (RMI2) to form a conserved Bloom syndrome (BS) complex, which is vital for maintaining genome stability [11]. BS and BS-like syndrome, characterized by genomic instability, are reported to be attributed to mutations in one of these four genes [12–14]. Biochemical data have implicated that the BS complex safeguards genome integrity by participating in HR repair [15, 16]. The HR repair pathway includes resection of DSB ends, RAD51 nucleoprotein fiber formation, D-loop formation, and double Holliday junction (dHJ) dissolution [17]. The BS complex is mainly involved in DNA end resection and dHJ dissolution [18, 19]. DSBs are recognized by the MRE11–RAD50–NBS1 (MRN) complex, which promotes the activation of ATM and retards the cell cycle progression [17]. Subsequently, the BS complex promotes the DNA end resection to prepare DNA for HR [20]. RAD51, a conserved recombinase, is loaded onto the resected 3’ overhang single-stranded DNA (ssDNA) to form a functional nucleoprotein filament capable of searching for and locating the homologous template to initiate DNA synthesis [21]. Finally, the BS complex dismantles the HR intermediate-dHJ to prevent crossovers [22, 23].

Structural and functional experiments demonstrated that RMI1 contained OB-fold (oligonucleotide/ oligosaccharide binding) domains to bind directly to Top3A and RMI2, which is critical for maintaining the stability of the BS complex [24, 25]. Our previous studies revealed the importance of RMI1 in response to replication stress induced by camptothecin and hydroxyurea [26, 27]. Yin et al. reported that RMI1 formed IR-induced nuclear foci at the cellular level [28]. However, few studies have reported on the role of RMI1 in response to IR-induced DNA damage and repair. Therefore, this study was performed to understand the cellular functions and molecular mechanism by which RMI1 maintains genomic stability after IR exposure. We demonstrated that the loss of RMI1 in cells resulted in slower proliferation and hypersensitivity to IR, enhanced levels of micronuclei and DNA damage, delayed cell cycle arrest, and defective HR repair, highlighting the importance of RMI1 in response to radiation damage.

## Results

### Silencing of RMI1 enhances cellular radiosensitivity

**Fig. 1:**
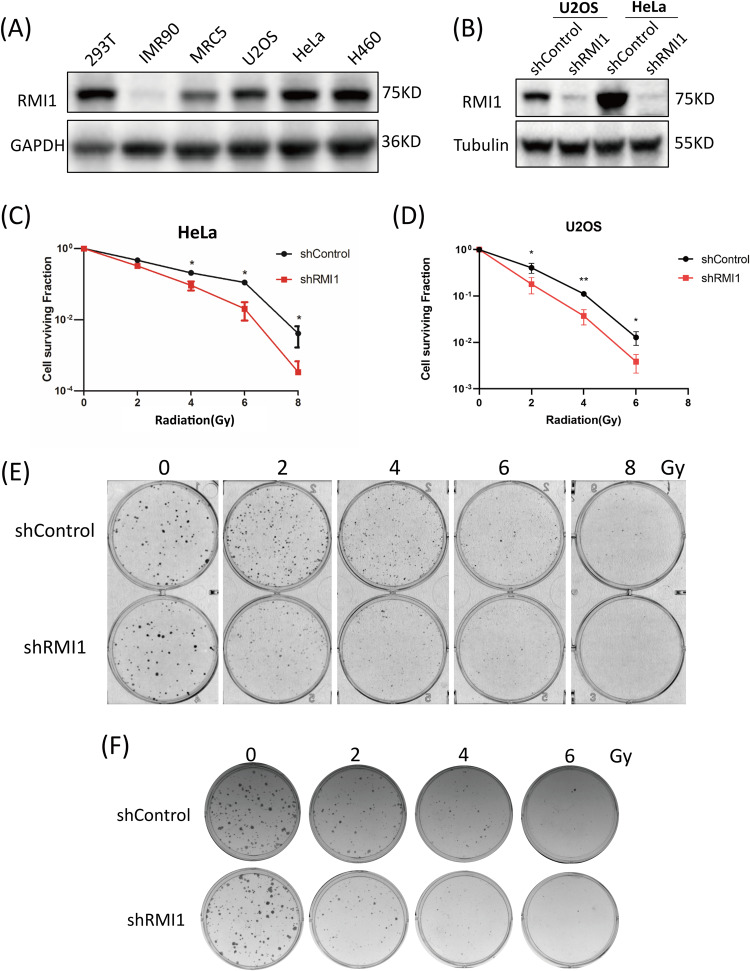
Silencing of RMI1 enhanced the radiosensitivity of HeLa and U2OS cells. **A** Expression analysis of RMI1 in human cell lines by western blot. **B** Western blotting of RMI1 in U2OS and HeLa cells infected with shControl or shRMI1 lentiviruses. **C**, **D** Clonogenic survival assay with HeLa and U2OS cells. Cells were exposed to increasing doses of irradiation, and the clonogenic survival was assessed after 10–14 days. **E**, **F** Representative diagram of cell colonies of HeLa and U2OS cells. (*P < 0.05, **P < 0.01).

To gain insight into the cellular function of RMI1 in response to IR, we first examined the protein levels of RMI1 in several human cell lines. As shown in Fig. 1A, the protein levels of RMI1 were relatively low in normal cell lines (IMR90 and MRC5) while relatively high in cancer cells (U2OS, HeLa, and H460), with the exception of 293 T cells. The protein level of RMI1 in 293 T cells was similar to that in cancer cells. Next, we investigated the cell proliferation in the RMI1-silenced cells after IR. We generated RMI1-silenced cells using short hairpin RNAs (shRNAs) delivered by lentivirus in MRC5 or 293 T cells, and the RMI1 depletion level was confirmed by western blotting (Supplementary Figs. S1A and S2A). Loss of RMI1 resulted in the markedly lower viability of MRC5 cells (Supplementary Fig. S1B). Compared with shControl and wild-type 293 T cells, RMI1-silenced cells exhibited a significantly lower proliferation ability, especially after IR treatment (Supplementary Fig. S2B–F). We further examined the effect of RMI1 silencing on radiosensitivity using the clonogenic survival assay in HeLa and U2OS cells. The lentiviral shRMI1 also markedly reduced the endogenous RMI1 protein levels in HeLa and U2OS cells (Fig. 1B). Consistently, RMI1 silencing led to decreased cell survival after IR (Fig. 1C–F). This finding indicates that RMI1-silenced cells exhibited more sensitivity to IR and suggests that RMI1 might protect cells from IR-induced DNA damage.

### RMI1 is a responsive protein to IR

**Fig. 2:**
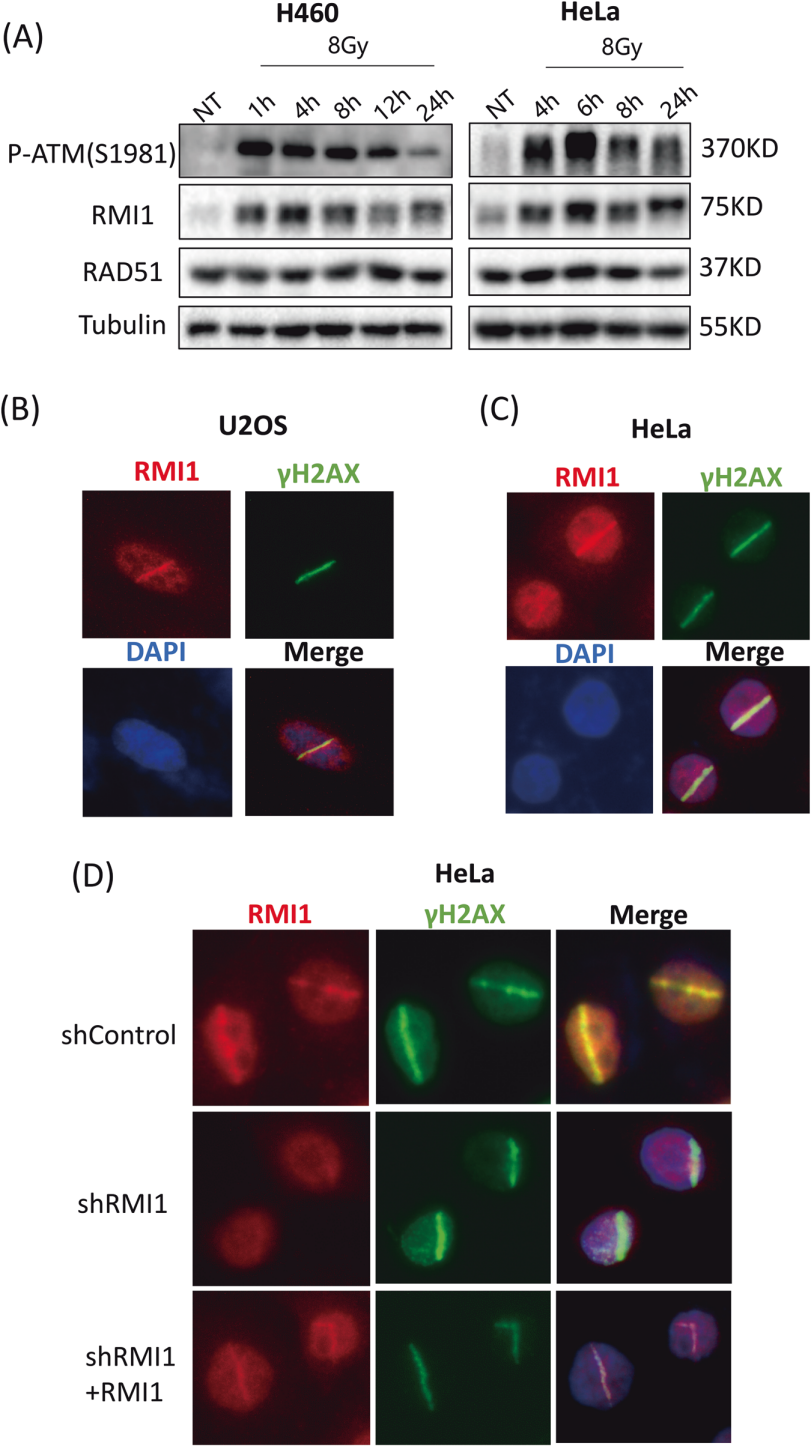
RMI1 is an IR-responsive protein. **A** Western blotting analysis of RMI1 protein in H460 and HeLa cells exposed to 8 Gy of γ-rays or not. **B**, **C** U2OS cells (**B**) and HeLa cells (**C**) were laser microirradiated and stained 1 h later for RMI1 and γH2AX. The green line (γH2AX) indicates the location of laser-induced DNA damage. **D** HeLa cells depleted of endogenous RMI1 (shRMI1) and complemented with RMI1 (shRMI1 + RMI1) were subjected to laser microirradiation. After 1 h, the cells were fixed and stained with anti-γH2AX and anti-RMI1 antibodies. 4’, 6-diamidino-2-phenylindole (DAPI) was used to stain the nucleus.

Given that RMI1 is important for cells to survive IR damage, we next sought to determine whether IR could affect RMI1 protein levels. As shown in Fig. 2A, we found that endogenous RMI1 protein levels were elevated after 8 Gy IR exposure in both H460 and HeLa cells, suggesting that RMI1 may respond to IR. Although it was reported that IR induced nuclear RMI1 foci formation [28], whether RMI1 can be recruited to sites of DNA lesions remains unclear. To address this, we induced DNA damage of single nuclei in both U2OS and HeLa cells by laser microirradiation. We found that endogenous RMI1 was recruited to these damage sites, where it co-localized with γH2AX (Fig. 2B, C). To confirm it, HeLa cells transduced with shControl or shRMI1 were also subjected to laser microirradiation. As shown in Fig. 2D, RMI1 depletion greatly diminished the accumulation of RMI1 at sites of laser-induced DNA damage, while the re-introduction of shRNA-resistant RMI1 into the RMI1-silenced HeLa cells (shRMI1 + RMI1) restored the localization of RMI1 at DNA damage sites.

### RMI1 maintains genome stability after radiation

**Fig. 3:**
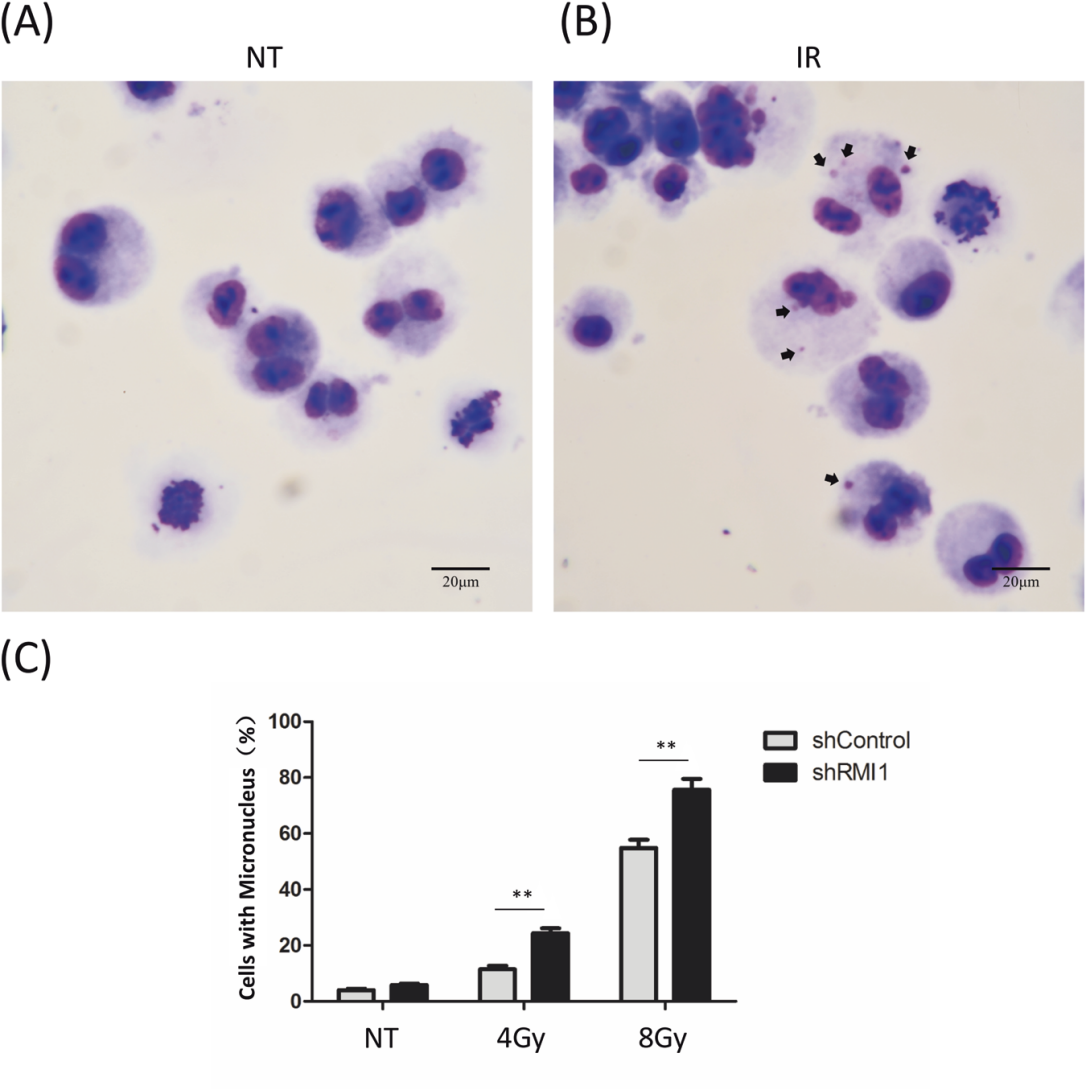
RMI1 silencing increased the micronucleus formation rate induced by IR. **A** Representative image of normal binuclear HeLa cells. **B** Representative image of binuclear HeLa cells containing micronuclei pointed by arrows. **C** Graph showing the percentage of cells with micronuclei. (**P < 0.01).

IR is known to induce micronuclei and chromosomal aberrations [29]. To examine the effect of RMI1 on genomic stability, HeLa shControl and shRMI1 cells were irradiated with 4 or 8 Gy of γ-rays, and the number of micronucleus in cytokinesis-blocked cells was detected. As shown in Fig. 3, the micronucleus formation rate of the shControl group was 13.2%, while that of the RMI1-silenced cells was 35.6% after treatment with 4 Gy irradiation. Similarly, a significantly higher level of micronuclei was shown in RMI1-silenced cells after treatment with 8 Gy irradiation, indicating that RMI1 silencing could increase the micronucleus formation rate after IR. In addition, we also examined the occurrence of DNA breaks, gaps, and dicentric and acentric fragment chromosomes in HeLa shRMI1 and shControl cells after IR exposure. Although metaphase spreads did not show significant differences in chromosome breaks, gaps, and acentric fragments between shRMI1 and shControl cells, a significantly higher incidence of dicentric chromosomes was observed in shRMI1 cells than in shControl cells 24 h after irradiation (Supplementary Fig. S3). These results indicated that the genomic instability increased in RMI1-silenced cells exposed to IR.

### RMI1 deficiency caused the accumulation of DNA damage

**Fig. 4:**
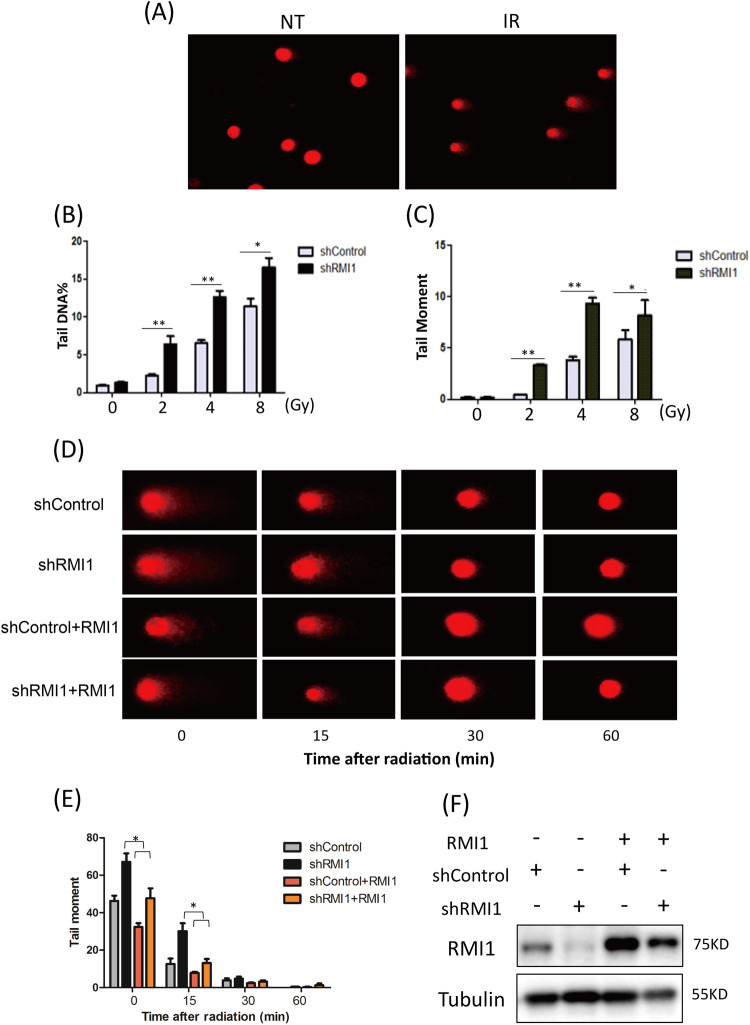
RMI1 deficiency aggravated IR-induced DNA damage. **A**–**C** HeLa shRMI1 and shControl cells were exposed to indicated doses of irradiation and the DNA damage level was determined using the comet assay. **A** Representative images of undamaged and damaged cells. Percentages of DNA tail (**B**) and tail moment (**C**) were analyzed. **D**, **E** HeLa shRMI1 and shControl cells transfected with RMI1-expressing plasmids or those not transfected were irradiated with 4 Gy of γ-rays and then subjected to the comet assay at indicated time points. **D** Representative images. **E** Tail moment was quantified and graphed. **F** Cells subjected to the comet assay were analyzed for RMI1 by Western blot. The level of DNA damage was represented as the mean of three independent experiments, and at least 200 cells were counted. (*P < 0.05, **P < 0.01).

Given that the formation of abnormal chromosomes is generally caused by unrepaired damaged DNA, we next explored whether RMI1 silencing led to more irradiation-induced DNA damage. To accomplish this, we used an alkaline comet assay. First, HeLa shControl and shRMI1 cells were collected immediately after 2, 4, and 8 Gy irradiation and total DNA lesions were assessed. We observed the increased percentage of DNA tails after treatment with different irradiation doses in the shRMI1 cells compared with the shControl cells (Fig. 4A–C). Then, we studied the effect of RMI1 silencing on DNA damage repair progression. Following treatment with 4 Gy of irradiation, HeLa shControl, and shRMI1 cells were collected 0, 15, 30, and 60 min after irradiation, and the level of DNA damage was detected. The damaged DNA was repaired rapidly with the extension of time after irradiation, and most of the broken DNAs were repaired at 60 min after irradiation. However, the shRMI1 cells displayed significantly increased DNA tail levels 0 and 15 min after irradiation. To verify the effect of RMI1 on DNA damage repair, we expressed the shRNA-resistant RMI1 in cells depleted of endogenous RMI1. We found that supplementation of RMI1 reduced the level of damaged DNA following IR (Fig. 4D–F), implying that RMI1 might play an important role in repairing irradiation-induced DNA damage.

### RMI1 silencing led to the accumulation of γH2AX and 53BP1 foci

**Fig. 5:**
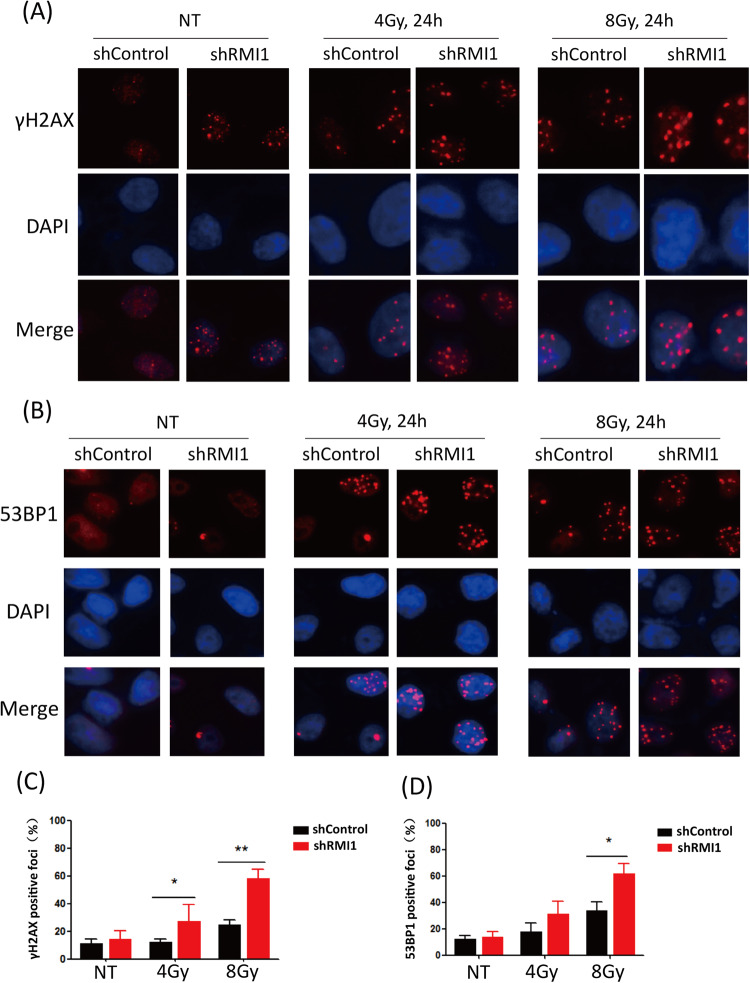
RMI1 silencing impaired the repair of radiation-induced DNA damage. **A** Representative images of γH2AX foci in HeLa shControl and shRMI1 cells. **B** Representative images of 53BP1 foci in HeLa shControl and shRMI1 cells. **C** γH2AX foci were counted 24 h after irradiation. **D** 53BP1 foci were counted 24 h after irradiation. The experiment was performed three times independently and more than 300 cells were counted for each group. (*P < 0.05, **P < 0.01).

To confirm the role of RMI1 in IR-induced DNA damage repair, we analyzed γH2AX and 53BP1 foci formation as the indicators of DNA damage using immunofluorescence. The levels of γH2AX and 53BP1 foci in HeLa RMI1-silenced cells were both significantly higher than those in control cells after 24-h exposure to 4 and 8 Gy of IR (Fig. 5), which was consistent with the results of comet assay. Likewise, there were more γH2AX foci in MRC5 RMI1-silenced cells than in control cells (Supplementary Fig. S4). It suggests that RMI1 silencing might cause more IR-induced DNA damage and decrease the DNA repair ability of cells.

### RMI1 promoted HR during DNA repair

**Fig. 6:**
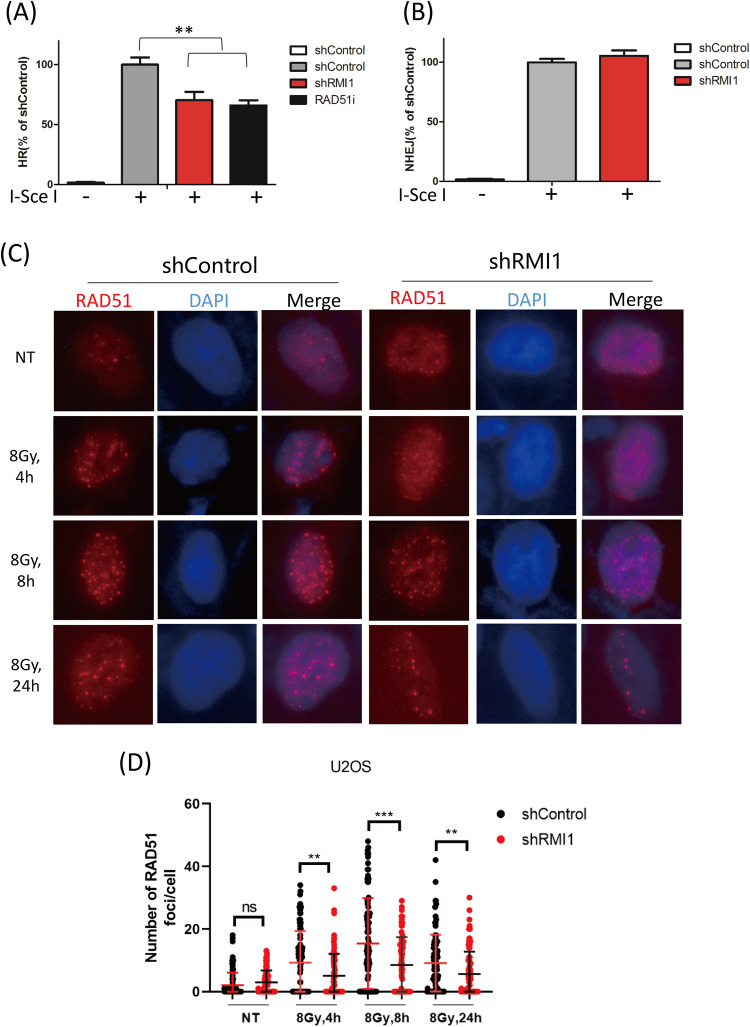
RMI1 promoted HR during DNA repair. **A** Flow cytometric analysis of HR efficiency in DR-GFP-U2OS cells. **B** Flow cytometric analysis of NHEJ efficiency in EJ5-GFP-U2OS cells. **C** Representative images of RAD51 foci in U2OS shControl and shRMI1 cells. **D** RAD51 foci were quantified. (**P < 0.01, ***P < 0.001).

NHEJ and HR are two major DNA double-strand break repair pathways [8]. To determine whether RMI1 would affect NHEJ and HR, we used EJ5-GFP-U2OS and DR-GFP-U2OS reporter cells to quantify NHEJ and HR efficiency in RMI1-silenced and control cells, respectively. As shown in Fig. 6A, the depletion of RMI1 significantly decreased the HR efficiency, and the cells treated with RAD51 inhibitors were used as a positive control. However, the rate of NHEJ remained unchanged after RMI1 silencing (Fig. 6B), suggesting that RMI1 could promote HR but not NHEJ during the repair of DSBs. RAD51 is a key recombinase involved in the HR process, and the presence of RAD51 foci serves as a functional marker for HR-mediated DNA repair. To further validate the function of RMI1 in promoting HR, we examined the RAD51 foci in RMI1-silenced and control cells after IR. Despite no obvious change in RAD51 protein level (Supplementary Fig. S5), substantially reduced RAD51 foci were observed in RMI1-silenced cells than in control cells after IR (Fig. 6C, D), corroborating the attenuated HR efficiency in RMI1 silencing.

### RMI1 loss intensified checkpoint response and G2 cell cycle arrest after irradiation

**Fig. 7:**
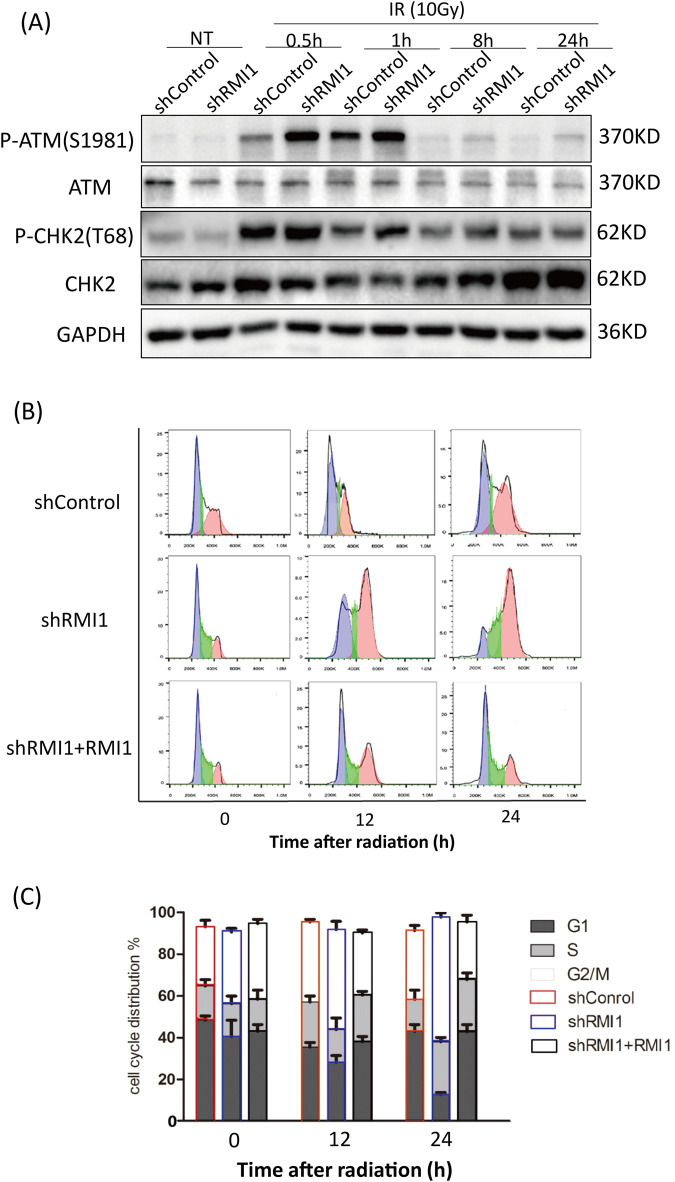
RMI1 influenced checkpoint activation and cell cycle arrest following radiation. **A** HeLa cells were harvested at indicated time points after 10 Gy of irradiation. Western blot analysis of phosphorylated and total protein levels of ATM and CHK2. GAPDH was used as a loading control. **B** Cell cycle distribution of HeLa cells was determined by flow cytometry 0, 12, and 24 h after irradiation. **C** Percentages of cell cycle phases are depicted.

To evaluate the effect of RMI1 on IR-induced DNA damage checkpoint activation and G2/M cell cycle arrest, we investigated whether RMI1 regulated the major kinase pathways ATM/CHK2. Western blot analysis showed that the phosphorylation of ATM and CHK2 increased more profoundly in RMI1-silenced cells than in control cells after irradiation (Fig. 7A). Further, we analyzed the cell cycle distribution using flow cytometry in HeLa shControl, shRMI1, and shRMI1 + RMI1 cells after IR. As shown in Fig. 7B, C, more RMI1-silenced cells were arrested in the G2/M phase than in the control group 12–24 h after IR treatment, and the reintroduction of RMI1 could alleviate the cell cycle arrest. These results demonstrated that RMI1 silencing caused stronger DNA damage checkpoint activation and more G2/M phase arrest in response to IR.

## Discussion

RMI1, an essential component of the BS complex, plays an important role in BLM-dependent genome maintenance [28]. Embryonic lethality of RMI1 knockout mice and RMI1 mutations identified in patients with BS-like disorder, highlighting the importance of RMI1 in embryonic development and preserving genome stability [12, 13, 30]. Our previous study found that the depletion of RMI1 in 293 T cells led to increased cell sensitivity and apoptosis following IR [31]. In our present study, we further investigated the roles of RMI1 in response to DNA damage induced by IR. In agreement with the previous study [28], we confirmed that RMI1 silencing inhibited the cell proliferation of MRC5 and 293 T cells without or with IR treatment (Supplementary Figs. S1 and S2). RMI1 silencing also made HeLa and U2OS cells hypersensitive to IR by detecting colony formation (Fig. 1), in accordance with our previous observation in 293 T cells [31]. To study the effect of RMI1 on genomic stability, we showed that RMI1 suppresses the incidence of micronuclei and chromosomal aberrations induced by IR (Fig. 3). Subsequently, the recruitment of RMI1 within laser-microirradiated regions suggested that RMI1 might function at DNA damage sites (Fig. 2). Then, we investigated whether RMI1 protected cells against IR via promoting DNA repair. We examined the IR-induced DNA damage using the comet assay. RMI1 silencing significantly increased the level of DNA damage and attenuated the DNA damage repair (Fig. 4). We further examined γH2AX and 53BP1 foci in control and RMI1-silenced cells and observed that RMI1 depletion increased the formation of γH2AX and 53BP1 foci (Fig. 5). The results indicated that RMI1 played an essential role in IR-induced DNA damage repair.

Cells have evolved complex mechanisms to protect against genomic instability, collectively called DNA damage response (DDR). DDR can detect broken DNA, activate the cell cycle checkpoint, block the cell cycle, and promote the repair of DNA damage [32]. ATM is a master regulator of cellular responses to DSBs, leading to the activation of a DSB-induced signaling cascade [33]. After DNA damage, CHK2 is phosphorylated by ATM on the priming site T68, which is routinely used to indicate ATM activation [34]. In this study, we found that RMI1 silencing aggravated the activation of cell cycle checkpoints, such as the elevated phosphorylation levels of ATM and CHK2 (Fig. 7A). The effect on cell cycle checkpoints seems to be dependent on the irradiation dose since the change of phosphorylated ATM and CHK2 after 4 Gy irradiation is not as obvious as that after 10 Gy irradiation (Supplementary Fig. S6). DDR is closely related to cell cycle progression, and many DDR proteins are regulated by the cell cycle. Therefore, the cell cycle distribution after IR was analyzed by flow cytometry. We found that RMI1 silencing caused more cell arrest in the G2/M phase, as shown in Fig. 7B, C. It implicated that loss of RMI1 impaired the DNA repair, resulting in sustained cell cycle arrest and intensified activation of DDR. This provided further evidence that RMI1 promoted IR-induced DNA damage repair.

Of the many different types of DNA damage induced by IR, the most harmful type is DSBs. In mammalian cells, DSBs are primarily repaired through NHEJ and HR [35]. To determine which repair pathway that RMI1 affects, we examined the repair efficiency of HR and NHEJ in RMI1-silenced cells. We found that RMI1 promoted HR, not NHEJ repair (Fig. 6A, B). Given that RAD51 nuclear foci is a hallmark for HR-mediated repair [36], we further explored how RMI1 influences HR efficiency by examining the RAD51 foci formation. We observed the impaired ability to form RAD51 nuclear foci in RMI1-silenced cells after IR treatment (Fig. 6C, D), suggesting that RMI1 might facilitate loading RAD51 to the DNA damage sites. These results were consistent with our previous findings of RMI1 functions in response to replication stress [26, 27]. The precise mechanism by which RMI1 facilitates the loading of RAD51 onto the DNA damage sites remains to be determined.

In conclusion, our study demonstrated that RMI1 was involved in the cellular response to IR via regulating HR-mediated DSB repair. Under the condition of IR damage, RMI1 deficiency resulted in enhanced levels of DNA damage, intensified DNA damage checkpoint activation, and retained cell cycle arrest. Furthermore, our findings revealed a failure to recruit RAD51 to the DNA breakage sites, resulting in insufficient HR, genomic instability, and loss of cell viability. These observations implied that RMI1 might be a new regulator for recruiting RAD51 to the sites of DSBs. Further studies are warranted to elucidate the detailed mechanism of RMI1 in response to IR.

## Materials and methods

### Cell culture and irradiation conditions

HeLa, U2OS, 293 T, MRC5, IMR90, and H460 cells were all purchased from the American Type Culture Collection (ATCC, VA, USA) and cultured with Dulbecco’s modified Eagle’s medium (Hyclone, UT, USA) supplemented with 10% FBS (Gibco, CA, USA) at 37 °C in a humidified incubator with 5% CO_2_. EJ5-GFP-U2OS and DR-GFP-U2OS cells were provided by Dr Xingzhi Xu (Shenzhen University, China). A ^137^Cs source (Canadian Atomic Energy Company, γ-Cell 40) was used for γ-ray irradiation, and the dose rate was 1 Gy/min.

### Lentiviral transductions and plasmid construction

The lentiviral transduction method was used as previously described [27]. The shRNA sequences were as follows: shRMI1#1: 5’-AGCCTTCACGAATGTTGAT-3’; shRMI1#2: 5’-GATCTAGTTACAGCTGAAG-3’. The shRNA-resistant RMI1 plasmid was constructed by site-directed mutagenesis of four nucleotides in the target sequence of shRMI1#1 as AGCCTAGCAGAATGTTGAT.

### Western blot analysis

The proteins from cells were extracted in RIPA lysis buffer with protease inhibitors on ice for 30 min. Total protein concentration was determined using a BCA protein assay kit (Beyotime Biotechnology, Shanghai, China), and the cell lysate was supplemented with 4× loading buffer and boiled. A total of 30 µg of protein from each sample was loaded onto SDS-PAGE and transferred onto an activated PVDF membrane (Millipore, MA, USA). The PVDF membranes were probed with primary antibodies overnight at 4 °C and with secondary antibodies for 1 h at room temperature. The primary antibodies used were as follows: RMI1 (14630-1-AP, Proteintech, Wuhan, China), phospho-CHK2 (Thr68) (#2661, Cell signaling technology, MA, USA), CHK2 (#3440, Cell signaling technology, MA, USA), phospho-ATM (Ser1981) (#13050, Cell signaling technology, MA, USA), ATM (#2873 S, Cell signaling technology, MA, USA), RAD51 (PC130, Millipore, MA, USA), Tubulin (66240-1-Ig, Proteintech, Wuhan, China), GAPDH (60004-1-Ig, Proteintech, Wuhan, China).

### CCK-8 and Celigo cell-counting assay

The cells in the logarithmic growth phase were used to prepare cell suspensions and counted. Then, the cells were plated in 96-well plates with 1000 cells (CCK-8 assay) or 200 cells (cell-counting assay) per well and incubated at 37 °C with 5% CO_2_. After 24 h of cultivation, cells were irradiated with the indicated doses. Forty-eight hours later, cells in each well were incubated with 10 μl of CCK-8 solution (Meilunbio, China) for 4 h before measuring the absorbance at 450 nm by a microplate reader (Thermo Fisher, MA, USA). For the cell-counting assay, cells were recorded by Celigo Imaging Cytometer (Nexcelom, MA, USA) once a day, and the plates were read for five consecutive days.

### Clonogenic survival assay

The cells were plated in triplicate into six-well plates and exposed to the indicated doses of γ-rays the next day. Then, the cells were cultured in a complete medium for 10–14 days. The surviving colonies were stained with 0.25% (w/v) crystal violet in ethyl alcohol for 15 min at room temperature, and the colonies consisting of more than 50 cells were counted.

### Analysis of micronuclei

The HeLa shControl and shRMI1 cells were treated with 4 and 8 Gy of IR, and the cultures were incubated at 37 °C for 24 h. Cytochalasin B was added for a final concentration of 6 mg/mL, and the cultures were then incubated for 44 h. The cells were harvested, fixed with methanol and acetic acid (3:1), dropped onto dry clean slides, and stained with 5% Giemsa (Sigma-Aldrich). For each slide, the micronuclei were scored in at least 1000 binucleated cells for each experimental point according to the criteria proposed by a Zeiss MetaSystem (Germany).

### Analysis of chromosomal aberrations

The HeLa shControl and shRMI1 cells were plated in six-well plates, exposed to 8 Gy irradiation, and then incubated with 5% CO_2_ at 37 °C for 48 h. Colchicine (RuiAiJin, Tianjin, China) was added to the culture medium for a final concentration of 0.06 μg/mL, and the cells were cultured for an additional 6 h. The cells were harvested for hypotonic treatment, fixed, spread onto slides, air-dried, and stained with Giemsa (Sigma-Aldrich). At least 100 metaphase cells with well-spread chromosomes were analyzed for each group.

### Alkaline comet assay

The comet assay was performed as previously described [26]. The cells were exposed to different radiation doses (2, 4, and 8 Gy) and incubated at 37 °C at different time points before collection. The cells were mixed with low-melting agarose and spread on fully frosted slides precoated with normal agarose. After solidification, the slides were immersed in lysis buffer for 2 h at 4 °C. After lysis, the slides were equilibrated and electrophoresed in 1× TAE buffer at 4 °C. The slides were stained with Gel-Red and visualized using a fluorescence microscope (ETLPSE 90i; Nikon, Tokyo, Japan). The fluorescence was recorded and analyzed using CaspLab Software (Wroclaw, Poland).

### Immunofluorescence

The cells were plated in 12-well plates with glass coverslips 24 h before IR treatment. The treated cells were fixed with 4% formaldehyde for 15 min, washed three times with phosphate-buffered saline (PBS), and then permeabilized using 0.3% Triton X-100. After blocking with 1% bovine serum albumin diluted with PBS for 1 h at room temperature, the cells were incubated with the following primary antibodies at 4 °C overnight: phospho-histone H2AX (Ser139) (05-636, Millipore, MA, USA); 53BP1 (ab36823, Abcam, Cambridge, UK); RAD51 (PC130, Millipore, MA, USA). Subsequently, the cells were incubated with secondary antibodies. The fluorescence was monitored using a fluorescence microscope (Thermo Fisher, MA, USA), and foci were quantified using ImageJ software.

### Laser microirradiation

Cells were cultured on LabTek II chamber slides (Thermo Fisher, MA, USA) one day before induction of DNA damage by a 355 nm UV-A laser using a Zeiss inverted microscope with a PALM MicroBeam laser microdissection workstation (Carl Zeiss, Jena, Germany). One hour later, the cells were fixed with 4% formaldehyde for 10 min at room temperature and followed by the standard immunostaining protocol. Primary antibodies, RMI1 (14630-1-AP, Proteintech, Wuhan, China) and phospho-histone H2AX (Ser139) (05-636, Millipore, MA, USA), were used and DAPI was counterstained to visualize nuclear DNA.

### NHEJ and HR assay

EJ5-GFP-U2OS cells were used to measure the NHEJ efficiency, whereas DR-GFP-U2OS cells were used to detect the HR efficiency. The two kinds of cells were infected with lentiviruses containing shControl or shRMI1, respectively. After verifying RMI1 silencing, the cells were transfected with I-Sce I expressing plasmids using Lipofectamine 2000 (Thermo Fisher, MA, USA). The cells were collected 48 h later, and the percentage of GFP-positive cells was detected using flow cytometry.

### Flow cytometric analysis of cell cycle

The cells were plated in six-well plates, exposed to 8 Gy irradiation, and harvested 12 and 24 h later. They were washed with PBS, fixed with chilled 70% ethanol, and stored at −20 °C overnight. They were washed twice with cold PBS and incubated with staining buffer (4 mM sodium citrate, 50 μg/mL propidium iodide, and 2 μg/mL RNase A) at 37 °C for 15 min in the dark. The cell cycle was examined by flow cytometry (BriCyte E6; Mindray, Shenzhen, China), and analyzed using FlowJo software (V7.6).

### Statistical analysis

Each experiment was performed independently at least three times, and the results were shown as mean ± standard error of the mean. Statistical analyses were performed using SPSS software (2.6.0) and the Prism software program (GraphPad 7 Software). Differences among variables were analyzed using the two-tailed Student *t*-test. Between groups, differences with *P*-values less than 0.05 indicated as statistically significant differences between groups (*P < 0.05, **P < 0.01, ***P < 0.001).

### Supplementary information

Revised Supplementary Figure

## Data Availability

All data are available in the main text or the Supplementary Materials.

## References

[1] Barlow JH, Nussenzweig A (2014). Replication initiation and genome instability: a crossroads for DNA and RNA synthesis. Cell Mol Life Sci.

[2] Ui A, Chiba N, Yasui A (2020). Relationship among DNA double-strand break (DSB), DSB repair, and transcription prevents genome instability and cancer. Cancer Sci.

[3] Chen Y, Cui J, Gong Y, Wei S, Wei Y, Yi L (2021). MicroRNA: a novel implication for damage and protection against ionizing radiation. Environ Sci Pollut Res Int.

[4] Baulch JE (2019). Radiation-induced genomic instability, epigenetic mechanisms and the mitochondria: a dysfunctional ménage a trois?. Int J Radiat Biol.

[5] Fang L, Li J, Li W, Mao X, Ma Y, Hou D (2019). Assessment of genomic instability in medical workers exposed to chronic low-dose X-rays in Northern China. Dose Response.

[6] Huang RX, Zhou PK (2020). DNA damage response signaling pathways and targets for radiotherapy sensitization in cancer. Signal Transduct Target Ther.

[7] Santivasi WL, Xia F (2014). Ionizing radiation-induced DNA damage, response, and repair. Antioxid Redox Signal.

[8] Zhang Z, Samsa WE, De Y, Zhang F, Reizes O, Almasan A (2023). HDGFRP3 interaction with 53BP1 promotes DNA double-strand break repair. Nucleic Acids Res.

[9] Wright WD, Shah SS, Heyer WD (2018). Homologous recombination and the repair of DNA double-strand breaks. J Biol Chem.

[10] Shibata A, Jeggo PA (2020). Roles for 53BP1 in the repair of radiation-induced DNA double strand breaks. DNA Repair (Amst).

[11] Manthei KA, Keck JL (2013). The BLM dissolvasome in DNA replication and repair. Cell Mol Life Sci.

[12] Gönenc II, Elcioglu NH, Martinez Grijalva C, Aras S (2022). Phenotypic spectrum of BLM- and RMI1-related Bloom syndrome. Clin Genet.

[13] Martin CA, Sarlós K, Logan CV, Thakur RS, Parry DA, Bizard AH (2018). Mutations in TOP3A cause a bloom syndrome-like disorder. Am J Hum Genet.

[14] Hudson DF, Amor DJ, Boys A, Butler K, Williams L, Zhang T (2016). Loss of RMI2 increases genome instability and causes a bloom-like syndrome. PLoS Genet.

[15] Wu L, Bachrati CZ, Ou J, Xu C, Yin J, Chang M (2006). BLAP75/RMI1 promotes the BLM-dependent dissolution of homologous recombination intermediates. Proc Natl Acad Sci USA.

[16] Hodson C, Low JKK, van Twest S, Jones SE, Swuec P, Murphy V (2022). Mechanism of Bloom syndrome complex assembly required for double Holliday junction dissolution and genome stability. Proc Natl Acad Sci USA.

[17] Sung P, Klein H (2006). Mechanism of homologous recombination: mediators and helicases take on regulatory functions. Nat Rev Mol Cell Biol.

[18] Gravel S, Chapman JR, Magill C, Jackson SP (2008). DNA helicases Sgs1 and BLM promote DNA double-strand break resection. Genes Dev.

[19] Wu L, Hickson ID (2003). The Bloom’s syndrome helicase suppresses crossing over during homologous recombination. Nature.

[20] Soniat MM, Nguyen G, Kuo HC, Finkelstein IJ (2023). The MRN complex and topoisomerase IIIα-RMI1/2 synchronize DNA resection motor proteins. J Biol Chem.

[21] Zhao W, Steinfeld JB, Liang F, Chen X, Maranon DG, Jian Ma C (2017). BRCA1-BARD1 promotes RAD51-mediated homologous DNA pairing. Nature.

[22] Raynard S, Bussen W, Sung P (2006). A double Holliday junction dissolvasome comprising BLM, topoisomerase IIIalpha, and BLAP75. J Biol Chem.

[23] Xue X, Raynard S, Busygina V, Singh AK, Sung P (2013). Role of replication protein A in double holliday junction dissolution mediated by the BLM-Topo IIIα-RMI1-RMI2 protein complex. J Biol Chem.

[24] Bocquet N, Bizard AH, Abdulrahman W, Larsen NB, Faty M, Cavadini S (2014). Structural and mechanistic insight into Holliday-junction dissolution by topoisomerase IIIα and RMI1. Nat Struct Mol Biol.

[25] Wang F, Yang Y, Singh TR, Busygina V, Guo R, Wan K (2010). Crystal structures of RMI1 and RMI2, two OB-fold regulatory subunits of the BLM complex. Structure.

[26] Fang L, Sun X, Wang Y, Du L, Ji K, Wang J (2019). RMI1 contributes to DNA repair and to the tolerance to camptothecin. FASEB J.

[27] Xu C, Fang L, Kong Y, Xiao C, Yang M, Du LQ (2017). Knockdown of RMI1 impairs DNA repair under DNA replication stress. Biochem Biophys Res Commun.

[28] Yin J, Sobeck A, Xu C, Meetei AR, Hoatlin M, Li L (2005). BLAP75, an essential component of Bloom’s syndrome protein complexes that maintain genome integrity. EMBO J.

[29] Durante M, Formenti SC (2018). Radiation-induced chromosomal aberrations and immunotherapy: micronuclei, cytosolic DNA, and interferon-production pathway. Front Oncol.

[30] Chen H, You MJ, Jiang Y, Wang W, Li L (2011). RMI1 attenuates tumor development and is essential for early embryonic survival. Mol Carcinog.

[31] Sun Y, Fang L, Yang M, He N, Wang J, Zhang M (2021). Identification and bioinformatic assessment of circRNA expression after RMI1 knockdown and ionizing radiation exposure. DNA Cell Biol.

[32] Ciccia A, Elledge SJ (2010). The DNA damage response: making it safe to play with knives. Mol Cell.

[33] Blackford AN, Jackson SP (2017). ATM, ATR, and DNA-PK: the trinity at the heart of the DNA damage response. Mol Cell.

[34] Zannini L, Delia D, Buscemi G (2014). CHK2 kinase in the DNA damage response and beyond. J Mol Cell Biol.

[35] Ali A, Xiao W, Babar ME, Bi Y (2022). Double-stranded break repair in mammalian cells and precise genome editing. Genes (Basel).

[36] Chopra N, Tovey H, Pearson A, Cutts R, Toms C, Proszek P (2020). Homologous recombination DNA repair deficiency and PARP inhibition activity in primary triple negative breast cancer. Nat Commun.

