# Supplementary material for: RMI1 facilitates repair of ionizing radiation–induced DNA damage and maintenance of genomic stability
Source: Cell Death Discov. 2023 Nov 25;9:426. doi: 10.1038/s41420-023-01726-1 (PMC10676437; doi:10.1038/s41420-023-01726-1)
Supplement: Supplementary file 1 — Revised Supplementary Figure [file 41420_2023_1726_MOESM1_ESM.pdf]

RMI1 facilitates repair of ionizing radiation-induced DNA damage and maintenance of genomic stability

Lianying Fang [1, 2,†], Yuxiao Sun [1,†], Mingxin Dong [1,†], Mengmeng Yang [1], Jianxiu Hao [1] , Jiale Li [1], Huanteng Zhang[1] , Ningning He [1,*] , Liqing Du [1,*] and Chang Xu [1,*]

1 Tianjin Key Laboratory of Radiation Medicine and Molecular Nuclear Medicine, Institute of Radiation Medicine, Chinese Academy of Medical Sciences and Peking Union Medical College, Tianjin 300192, China

2 School of Preventive Medicine Sciences, Institute of Radiation Medicine, Shandong First Medical University, Shandong Academy of Medical Sciences, Jinan 250062, China

* Correspondence: Heningning@irm-cams.ac.cn (N.H.); dlq@irm-cams.ac.cn (L.D.); xuchang@irm-cams.ac.cn (C.X.); Tel.: +86-22-85682371(N.H., L.D., and C.X.)

† These authors contribute equally to this work.

Supplementary Figures

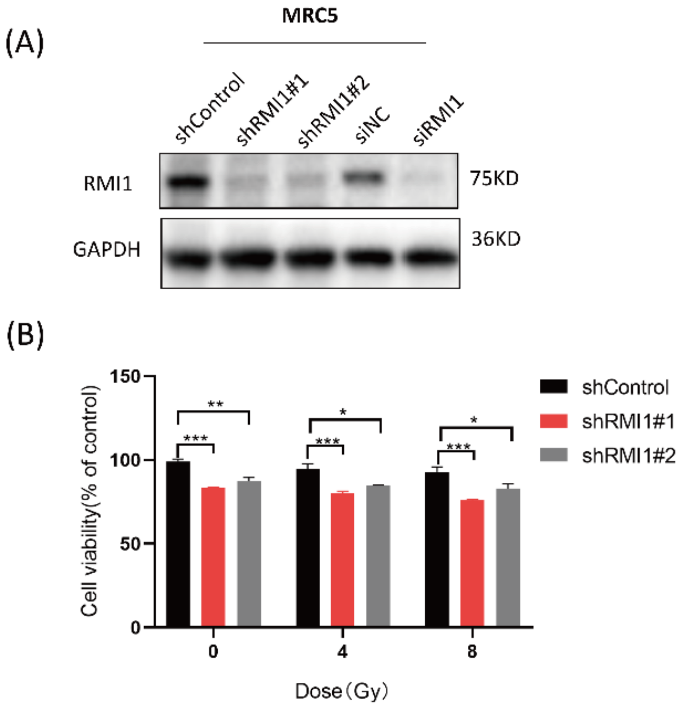

Figure S1. Silencing of RMI1 compromised the viability of MRC5 cells. (A) MRC5 cells were transfected with the indicated siRNAs or infected with lentiviruses expressing shControl, shRMI1#1, or shRMI1#2, and analyzed by western blot. (B) MRC5 cells with RMI1-silencing or not were exposed to different doses of irradiation and cell viability was measured by CCK-8 assay. (**P <0.05, **P < 0.01, ***P < 0.001*)

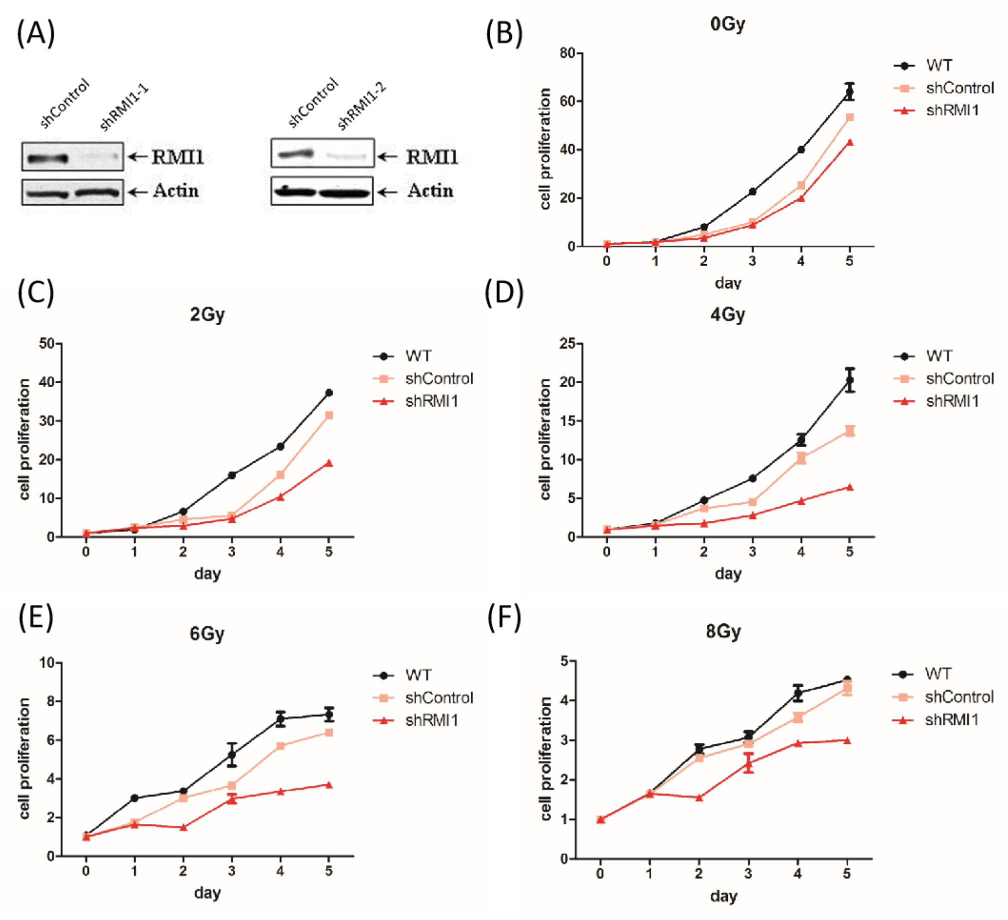

Figure S2. RMI1 silencing compromised the viability of 293T cell after exposure to IR. (A) Western blotting of RMI1 in 293T cells infected with lentiviruses expressing shControl, shRMI1-1, or shRMI1-2. (B-F) The cells (WT, shControl, shRMI1) were exposed to increasing doses of irradiation and cell viability was determined using Celigo Imaging Cytometer.

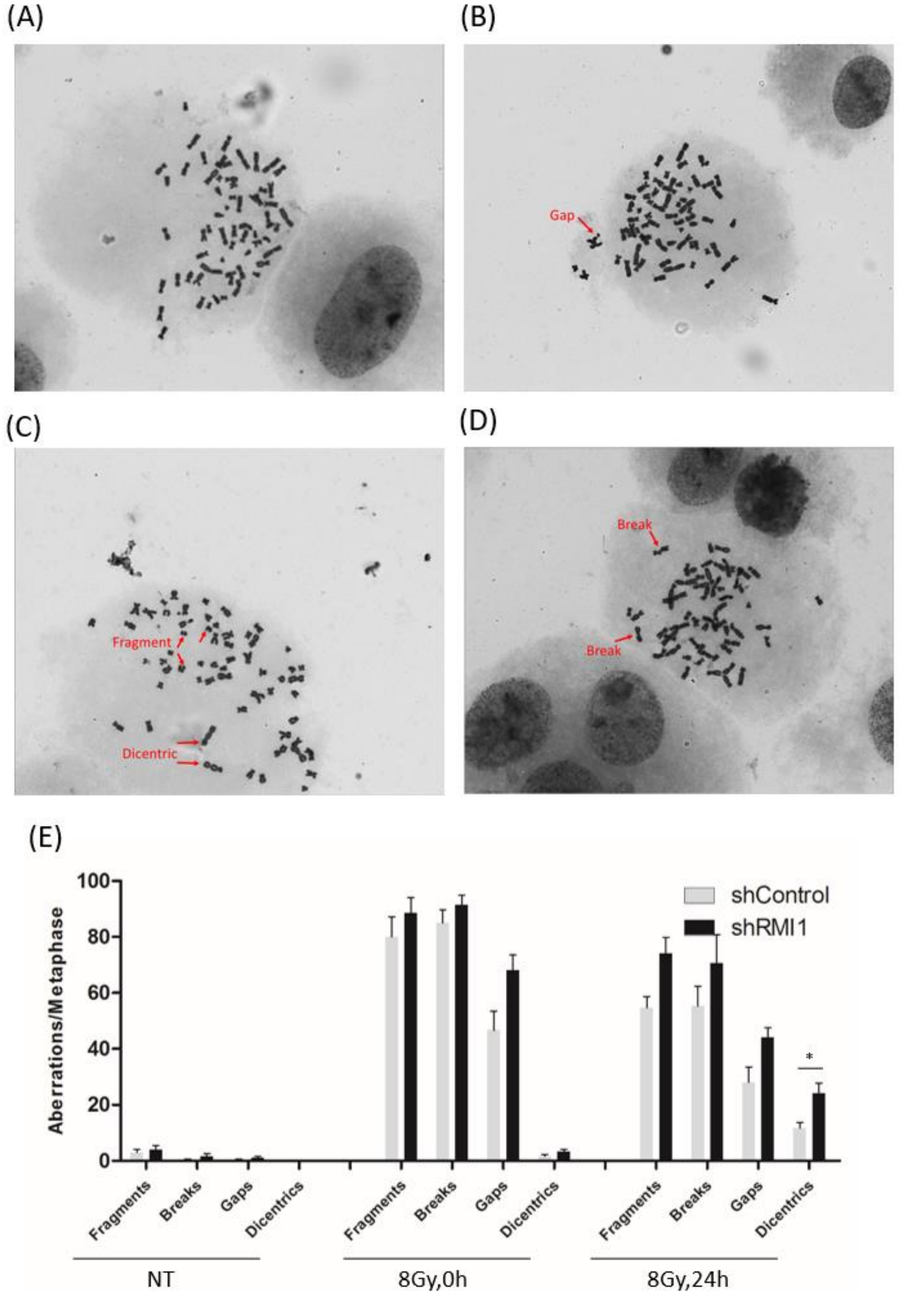

Figure S3. RMI1 silencing increased chromosomal aberration induced by IR. Representative image of normal chromosome (A), gap (B), dicentrics (C) and breaks (D). (E) Chromosomal aberrations in shControl or shRMI1 cells were quantitated. (*$P < 0.05$)

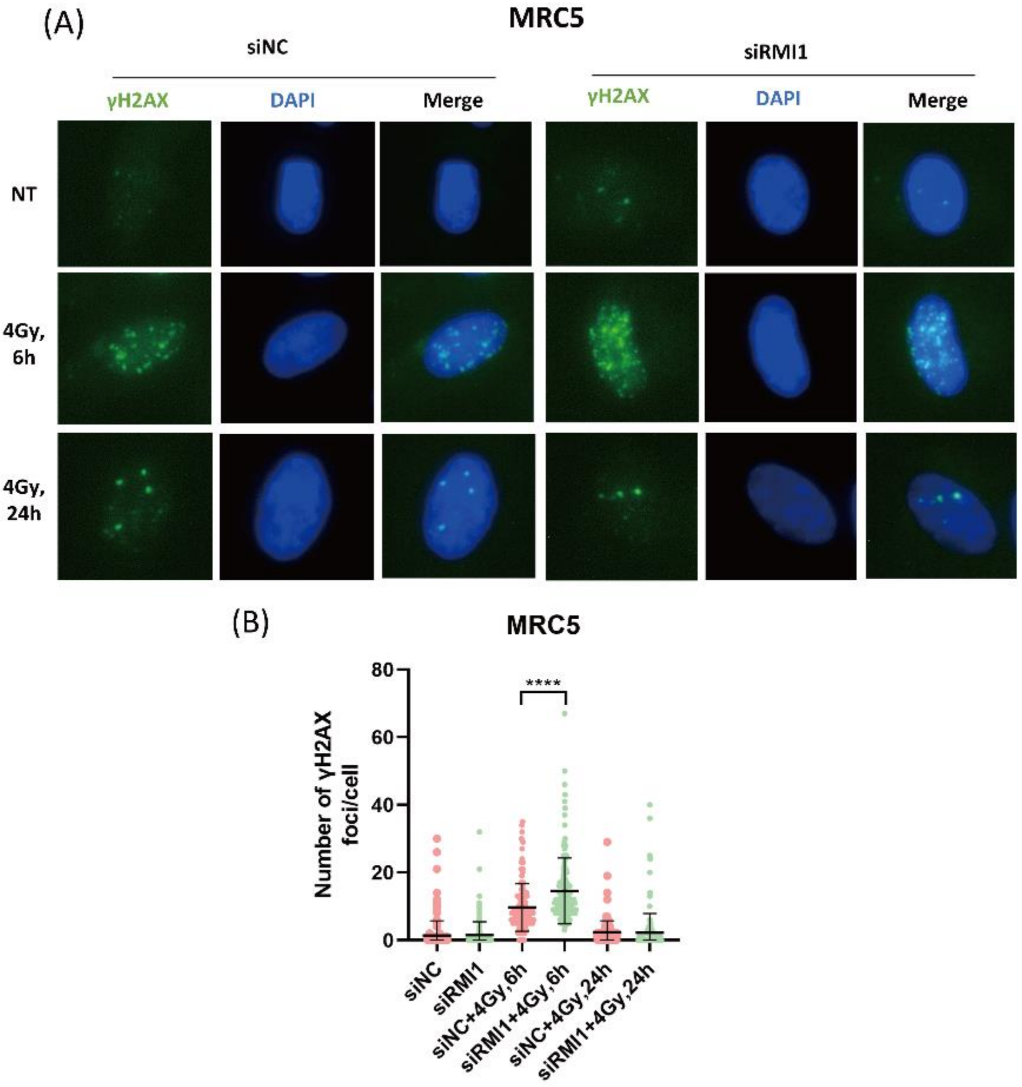

Figure S4. RMI1 silencing impaired the repair of radiation-induced DNA damage. (A) Representative images of γH2AX foci in MRC5 cells treated with siNC or siRMI1. (B) γH2AX foci numbers in each cells were quantified. (****$P < 0.0001$).

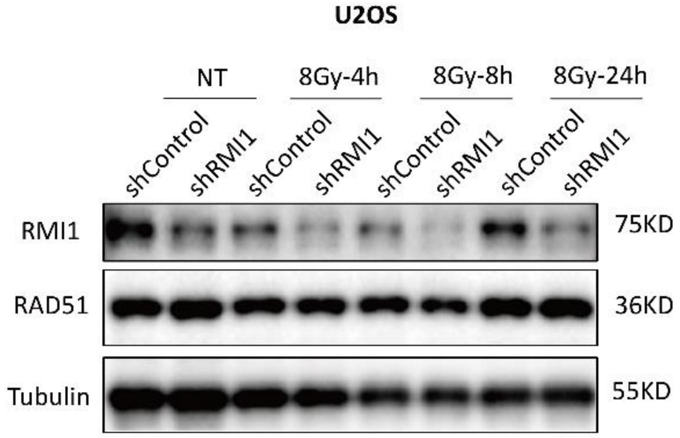

Figure S5. Western blot analysis of RAD51 in U2OS shControl and shRMI1 cells after irradiation.

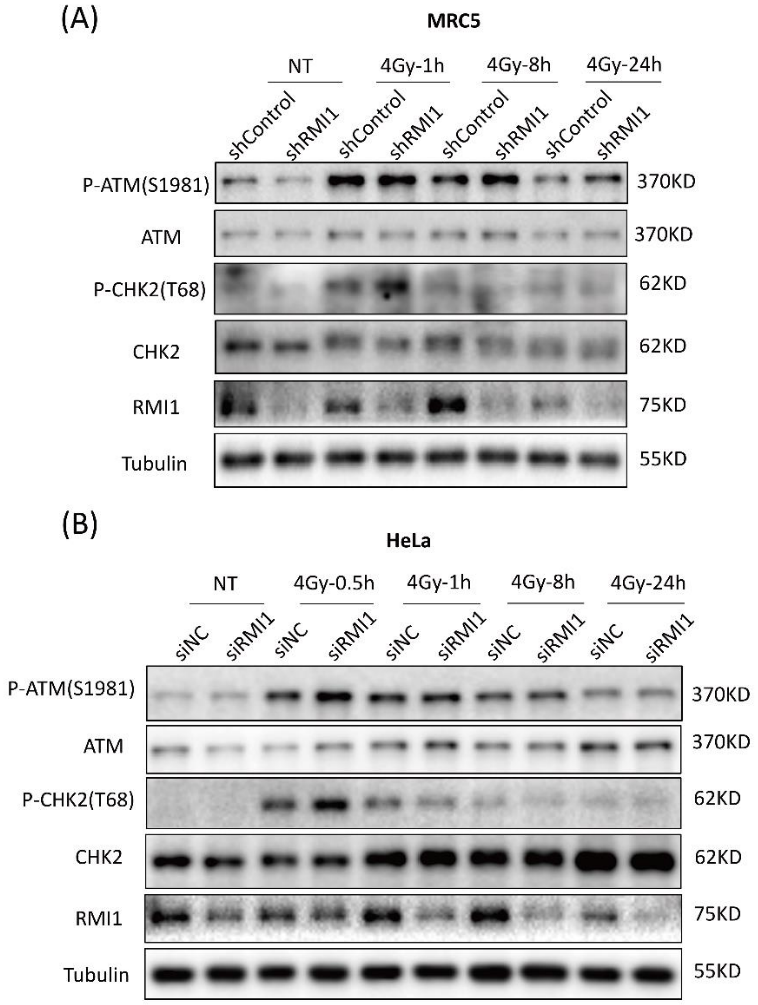

Figure S6. RMI1 silencing affected DNA damage checkpoint activation. MRC5 cells (A) and HeLa cells (B) were harvested at indicated time points after 4 Gy of irradiation and blotted with the indicated antibodies.
